# Oncologic First Events in Breast Cancer Patients After Targeted Axillary Dissection

**DOI:** 10.1245/s10434-025-18068-0

**Published:** 2025-08-20

**Authors:** Frederikke Munck, Maj-Britt Jensen, Riazan Hawaz-ali, Tove H. F. Tvedskov

**Affiliations:** 1 Department of Breast Surgery, Herlev-Gentofte Hospital, Hellerup, Denmark; 2 Danish Breast Cancer Group, Copenhagen University Hospital–Rigshospitalet, Copenhagen, Denmark

**Keywords:** Breast cancer, Neoadjuvant treatment, Targeted axillary dissection, Oncological outcomes, Regional nodal recurrence

## Abstract

**Background:**

Currently, targeted axillary dissection (TAD) is used in many centers as an alternative to axillary dissection for patients with lymph node metastases at diagnosis who receive neoadjuvant treatment. This allows patients to benefit from an axillary pathologic complete response rate as high as 60% during neoadjuvant treatment. Although the false-negative rate for TAD is low, data on oncologic outcomes are sparse and with limited follow-up time only. The study aimed to determine regional nodal recurrence and overall survival in a cohort of patients receiving TAD after neoadjuvant treatment.

**Methods:**

Prospective follow-up data on cN1-3 breast cancer patients receiving TAD with ypN0 lymph nodes after neoadjuvant treatment were collected from the Danish Breast Cancer Group Database. The patients received surgery between 2016 and 2021. No patients had completion axillary dissection. Data were left-truncated and analyzed with the cumulative incidence function and competing risk approach and Kaplan-Meier methods. The main outcomes were 5-year regional nodal recurrence and overall survival.

**Results:**

Among 283 patients with ypN0 status when staged by TAD, the 5-year regional nodal recurrence rate was 1.1% (95% confidence interval [CI], 0.30–2.9%). This corresponded to three regional nodal recurrence events, in which all patients had synchronous distant metastases. The 5-year overall survival rate was 95.1% (95% CI 92.4–98.0%).

**Conclusions:**

The low rate of regional nodal recurrence suggests effective long-term regional control and good overall survival for patients receiving TAD without axillary lymph node dissection.

**Supplementary Information:**

The online version contains supplementary material available at 10.1245/s10434-025-18068-0.

In recent years, minimally invasive axillary staging surgery has gained acceptance for patients who have node-positive breast cancer treated with neoadjuvant chemotherapy (NACT). Up to 60% of patients receiving NACT can achieve axillary pathologic complete response, depending on the receptor profile of the tumor.^1^ Use of less invasive staging methods allows patients to benefit from attaining an axillary pathologic complete response by avoiding an axillary lymph node dissection (ALND), a procedure known to carry risks such as lymphedema, pain, paresthesia, and functional impairment.^2,3^

Currently, sentinel lymph node biopsy (SLNB) is used for axillary staging after NACT in some breast surgery centers, provided the sentinel node (SN) yield is sufficient to ensure a satisfactory false-negative rate (FNR).^4,5^ An alternative to SLNB is targeted axillary dissection (TAD), in which SLNB is combined with targeted excision of a lymph node marked before NACT. Many studies have since investigated the feasibility and accuracy of TAD using various marking methods. In 2021, a meta-analysis demonstrated a pooled FNR for TAD of 5.2%.^6^

Refraining from ALND for patients with axillary pathologic complete response when staged by SLNB or TAD should not cause prognosis deterioration. Currently, data on oncologic outcomes according to axillary staging procedure are sparse. Additionally, no threshold for acceptable oncologic safety has been set.

Since 2016, TAD has been included in the Danish national guideline as a staging procedure for patients with cN1-3 disease at diagnosis who receive NACT. The procedure has been simultaneously implemented in all breast surgery departments.^7^ The early adoption of TAD enables us to report oncologic outcomes with 5 years of follow-up evaluation for a consecutive and national cohort of patients staged by TAD dating back to the introduction of TAD in Denmark. This study analyzed regional nodal recurrence (RNR) and overall survival for patients staged by TAD who achieved an axillary pathologic complete response.

## Methods

### Patient Selection

Danish patients with cN1-3 breast cancer verified on biopsy from lymph nodes and staged by TAD after NACT between 1 January 2016 and 31 August 2021 were enrolled in previously described retrospective national cohorts used for TAD feasibility studies and nodal burden prediction.^8-10^ The study included only patients who received TAD without completion ALND and had pathologic complete response in the TAD lymph nodes.

All the patients received surgical treatment at one of nine breast surgery departments in Denmark. Data for the study came from the Danish Breast Cancer Group (DBCG) database, which prospectively collects data on oncologic events for Danish patients with breast cancer. Clinical N stage is not registered in the DBCG database. Therefore, clinical N stage was searched in patient files. However, due to insufficient or missing description of the clinical N stage on ultrasound, imaging could not be used to identify patients with cN2-3 diseases. Instead, registration in the DBCG database with matted axillary lymph nodes, infraclavicular or supraclavicular metastases, or malignant tumor cells from infra- or supraclavicular nodes was used as a definition for the cN2-3 status.

A cohort of patients with cN0 breast cancer included between 2012 and 2021 who had received upfront surgery and axillary staging with SLNB and had a negative SN and no ALND was chosen for reference.

### The Danish Breast Cancer Group Database

Information on the diagnosis, treatment, and follow-up evaluation of all patients with primary breast cancer in Denmark is prospectively registered in the DBCG database by data submission from all departments involved in breast cancer care. Ethnicity data are not registered in the database and therefore were not collected in this study.

After a primary breast cancer, patients are followed for 10 years or until recurrence with either planned visits or patient-controlled follow-up evaluation.^11^ Patients exempt from systematic follow-up evaluation are those with a history of malignancy, bilateral breast cancer, inoperable breast cancer at diagnosis, or a contraindication to the recommended surgical procedures. The DBCG database is linked to the Danish Civil Registration System^12^ for a complete registration of vital status.

### Exclusion Criteria, Definitions, and Censoring

The study excluded patients with previous malignancies or missing follow-up data. To account for condition pre-selection in the TAD cohort, follow-up evaluation of the patients in both the study group and the reference group was left-truncated at day 201 after diagnosis, corresponding to the third quartile of the time between diagnosis and surgery in the study cohort.

Survival status and information on recurrence were collected on the patients in both groups. The definition of RNR followed the Standardized Definitions for Efficacy End Points (STEEP) criteria.^13^ For the calculation of RNR, the patients were censored at the last known follow-up day. Overall survival time was defined as the time from surgery to death from any cause, and the patients were censored if no event had occurred at the last synchronization date between the DBCG database and the Danish Civil Registration System.

### Adjuvant Radiotherapy

All the patients receiving axillary staging with TAD but no completion ALND are recommended to receive postoperative locoregional radiotherapy with a field that covers axillary levels 1 to 3, the supraclavicular, internal mammary, and interpectoral lymph nodes (Rotter’s nodes), and residual breast tissue or thoracic wall as appropriate.^14^ For the patients in the reference group who were cN0 and had pN0 status when staged by SLNB at upfront surgery, the contemporary recommendation was postoperative locoregional radiotherapy for those who had breast-conserving surgery or mastectomy for a T3-T4 tumor.^15,16^

### Statistical Analysis

The primary outcomes of the study were RNR and overall survival. Estimation of RNR was performed using the cumulative incidence function and a competing risk approach. Competing events were local relapse, distant relapse, contralateral breast cancer, other malignancies, and death as the first event. Overall survival was estimated using the Kaplan-Meier method. The reported follow-up times represent the estimated potential median follow-up time.^17^ The estimates were reported with a two-sided 95% confidence interval (CI). The baseline characteristics between the groups were compared with Pearson’s chi-square test or Fisher’s exact test as appropriate. The level of significance was set at 0.05. All analyses were performed using R statistical software (R Core Team, 2021, Vienna, Austria).^18^

The Legal Department of the Capital Region (j.no. p-2023-14336) approved the study’s data handling. The Centre for Health, Capital Region, Team for Medical Records Research (R-23038259) approved the study, waiving informed consent. The DBCG Steering Board granted permission for data retrieval from the DBCG database.

## Results

**Fig. 1:**
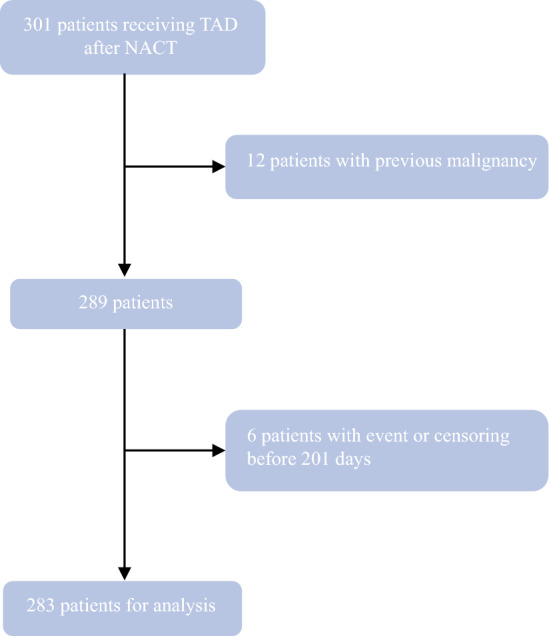
Exclusion and truncation in a cohort of 283 patients with ypN0 status at targeted axillary dissection (TAD) without completion axillary lymph node dissection (ALND)

The TAD cohort consisted of 301 patients, 12 of whom were excluded due to a medical history of previous malignancies. Thus, 289 patients were eligible, and after left-truncation of patients with an event or censoring before 201 days, 283 patients were eligible for analysis. Figure 1 presents the details of exclusion and truncation.

Most of the patients receiving TAD were younger than 70 years at surgery (86.5%; Table 1). In 13.8% of the patients, the breast tumor was 5 cm or larger before NACT, and 47% had estrogen receptor-negative (ER−) disease, whereas 64.7% had human epidermal growth factor receptor 2-positive (HER2+) disease. Mastectomy was performed for 38.9% of the patients. In 3.9%, cN2-3 disease was found.

**Table 1:** Characteristics of 283 ypN0 patients staged with TAD after NACT

	*n* (%)
*Age (years)*	
< 30	7 (2.5)
30–39	38 (13.4)
40–49	89 (31.4)
50–59	72 (25.4)
60–69	39 (13.8)
70–79	36 (12.7)
≥80	2 (0.7)
*Tumor size at ultrasound diagnosis (mm)*	
<20	63 (22.2)
≥20 to <50	181 (64.0)
≥50	39 (13.8)
*Clinical nodal stage*	
cN1	272 (96.1)
cN2-3	11 (3.9)
*Estrogen receptor status*	
Positive	150 (53.0)
Negative	133 (47.0)
*HER2 receptor status*	
Positive	183 (64.7)
Normal	100 (35.3)
*Breast surgery*	
Mastectomy	110 (38.9)
Breast-conserving surgery	173 (61.1)
*Breast tumor response*	
Pathologic complete response in breast	213 (73.7)
Residual tumor in breast	74 (25.6)
Missing	2 (0.7)
*Year of diagnosis*	
2016–2017	38 (13.4)
2018–2019	116 (41.0)
2020–2021	129 (45.6)

TAD, targeted axillary dissection; NACT, neoadjuvant chemotherapy; HER2, human epidermal growth factor receptor 2

**Fig. 2:**
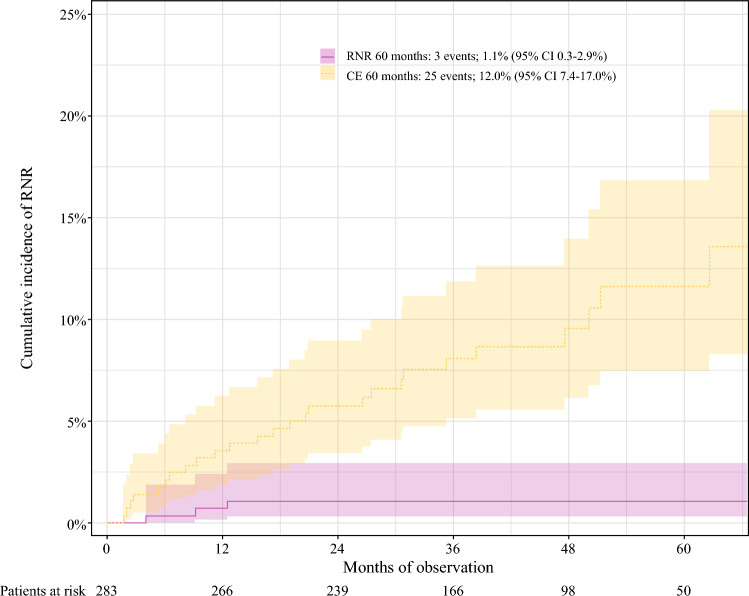
Cumulative incidence of regional nodal recurrence (RNR) and competing events (CE) in a cohort of 283 ypN0 patients staged with targeted axillary dissection (TAD) with no completion axillary lymph node dissection (ALND)

**Fig. 3:**
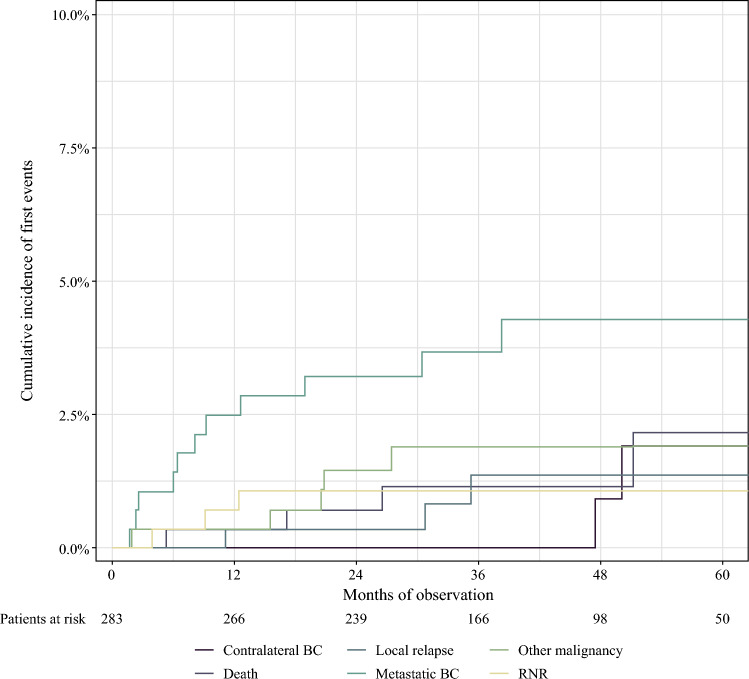
Cumulative incidence of 28 first events occurring in 283 ypN0 patients staged with targeted axillary dissection (TAD) after neoadjuvant chemotherapy (NACT) during a 5-year follow-up period

At the 5-year follow-up evaluation, only three patients had experienced an RNR. All three patients had synchronous distant metastases. None of these patients had a cN2-3 at diagnosis. The estimated potential median follow-up period was 42.1 months (IQR, 31.4–56.0 months). The 5-year cumulative incidence of RNR was 1.1% (95% CI 0.3–2.9%), whereas the 5-year rate of competing events was 12.0% (95% CI 7.4–17.0%) (Fig. 2). Figure 3 depicts the distribution of oncologic events in the TAD group.

**Fig. 4:**
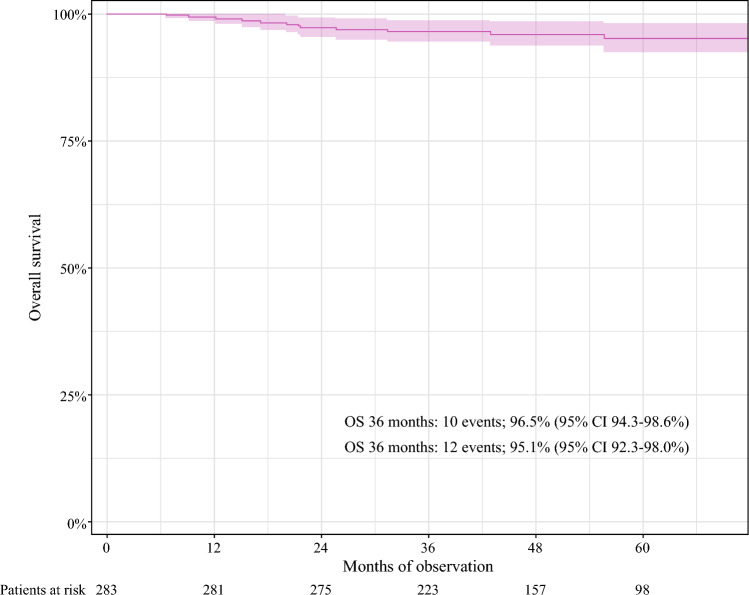
Overall survival (OS) in a cohort of 283 ypN0 patients staged with targeted axillary dissection (TAD) with no completion axillary lymph node dissection (ALND)

The overall survival analysis had an estimated potential median follow-up period of 52.2 months (IQR, 39.3–67.6 months). At the 5-year follow-up evaluation, 12 events had occurred, yielding a 5-year overall survival rate of 95.1% (95% CI 92.4–98.0%; Fig. 4).

The node-negative reference cohort consisted of 17,534 patients, 1154 of whom were excluded because they had a history of previous malignancies, leaving 16,380 patients eligible for the analysis. After left truncation, 15,784 patients remained for analysis. The details of exclusion in the node-negative cohort are shown in Figure, Supplemental Digital Content 1.

As expected, the node-negative cohort differed significantly from the TAD cohort by having more favorable prognostic characteristics (Table, Supplemental Digital Content 2 shows the characteristics of the node-negative cohort). In the node-negative cohort, 1.2% of the patients had tumors 5 cm or larger at diagnosis, 89.3% had ER+ disease, and 90.1% had HER2− disease. The patients in the node-negative cohort were older than those in the TAD cohort, with 74.9% younger than 70 years at diagnosis (25%).

At the 5-year follow-up evaluation, 92 patients in the node-negative cohort had an RNR. Among these patients, 48 had isolated RNR, 15 had synchronous local relapse, and 29 had synchronous distant metastases. The estimated potential median follow-up period for RNR was 72.1 months (IQR, 43.5–104.9 months). The resulting 5-year cumulative incidence of RNR was 0.69% (95% CI 0.56–0.84), and the competing events rate was 11.58% (95% CI 11.02–12.14%) (Figure, Supplemental Digital Content 3 shows RNR and competing event rates). Distribution of the events is shown in Figure, Supplemental Digital Content 4, which shows the cumulative incidence of oncologic first events in the node-negative cohort.

The 5-year overall survival rate was 93.9% (95% CI 93.5–94.3%) in the node-negative cohort, with an estimated potential median follow-up period of 93.5 months (IQR, 63.4–121.9 months) (Figure, Supplemental Digital Content 5 shows the overall survival rate in the node-negative cohort).

## Discussion

This study showed a very low 5-year RNR rate for the patients staged by TAD with ypN0 lymph nodes and no completion ALND. The RNR rate of 1.1% was higher than the RNR rate of 0.69% observed for the patients receiving upfront surgery with pN0 SNs and no ALND. However, the clinical significance of this difference seems negligible. A high overall survival rate was observed in the TAD cohort, exceeding 95% at 5 years, consistent with other studies showing the prognostic benefit of achieving an axillary pathologic complete response.^19,20^

A closer look at the patients in the TAD cohort who experienced an oncologic event showed that all the patients with RNR had synchronous distant metastases. This may raise the question whether these regional relapses are clinically important. However, the discovery of synchronous distant metastases in the presence of an RNR may be increased due to national guidelines recommending a positron emission tomography–computed tomography (PET–CT) as part of the diagnostic workup for all patients with a locoregional relapse.^21^

Only 3.9% of the patients were identified as having cN2-3 disease. None of these patients were among those who had an RNR, indicating that TAD could be a suitable staging procedure for this patient group. However, only a few patients were identified as having cN2-3 disease, and clinical N stage based on imaging could not be used. Further studies on this group of patients with advanced nodal stage are needed to fill the broad knowledge gaps, but more will be known from the ongoing Memorial Sloan Kettering Cancer Center (MSKCC) study of SLNB for patients with advanced nodal stage.^22^ However, guidelines for precise and uniform registration of clinical N stage at imaging also are needed for future studies on de-escalation of axillary treatment in this group of patients.

To place the RNR rate after TAD into a clinical perspective, we also reported the RNR rate for a cohort of cN0 and pN0 patients who underwent upfront surgery with SLNB and no completion ALND during the same period. For this group, the omission of ALND has long been accepted. This group had significantly better prognostic characteristics than the group receiving TAD. However, only a slight difference was seen in the RNR rate, and the group of patients receiving TAD had a comparable 5-year overall survival. Due to obvious differences in treatment and prognostic characteristics between the groups, a direct comparison was challenging. The most optimal setup would arguably have been a randomized trial comparing oncologic outcomes between the patients who were ypN0 when staged with TAD and those randomized to ALND or no ALND. However, such a trial would have required a very long follow-up period due to an expected low number of events and was not ethically justifiable at this stage, whereas TAD alone for patients with axillary pathologic complete response has been the standard treatment for several years in Denmark. We therefore chose a reference cohort of patients who were pN0 at staging for whom the omission of ALND is standard of care. Their favorable prognosis should ideally be emulated by patients receiving TAD.

To our knowledge, this is the first study to report both RNR and overall survival with a follow-up period up to 4.5 years after staging with TAD and no ALND. To date, few series, all with limited follow-up time, have been published on oncologic outcomes after minimal invasive axillary staging for patients with positive nodes before NACT. In the SENTA study, 119 patients received TAD, and axillary recurrences were observed in 1.8% of the patients during 3 years.^23^ The rate of ypN0 in the SENTA study was 79%.^23^ In a recent Turkish study of patients receiving TAD, a similarly low rate of regional recurrences (0.3%) after TAD is reported after a 3-year follow-up period.^24^ However, only 40% of their cohort attained an axillary pathologic complete response. Montagna et al.^25^ reported on a substantial cohort of ypN0 patients staged with either TAD or SLNB. The rate of axillary recurrences among the 478 patients with TAD at 3 years was 0.5%. Finally, the MARI protocol, in which 37% of 272 patients achieved an axillary pathologic complete response, reported a 1.8% axillary recurrence rate after a 3-year follow-up period (55 patients received completion ALND).^26^ Overall, these studies show a very low RNR rate for patients staged with minimally invasive axillary surgery after NACT when ALND is omitted in case of axillary complete response. Our study of patients staged by TAD currently confirms these results with a longer follow-up period.

It is important to note that all patients with pN^+^ breast cancer in Denmark are recommended to receive radiotherapy with a field that includes the axilla. This may have contributed to regional disease control. In the pursuit of safe de-escalation of regional therapy, results from the ongoing NSAPB-B51 trial will contribute important knowledge regarding the necessity of regional nodal radiotherapy for patients with verified lymph node-positive disease at diagnosis who attain ypN0 status at axillary staging.^27^

Compared with other studies, our study reported follow-up evaluation for an ample cohort of patients receiving TAD after NACT with ypN0 status and thus no ALND. We present these data together with data from a patient group with a known favorable prognosis. Despite the prognostic differences, the two groups had a very low RNR rate at this point. This provides a strong argument for safe regional control when ALND is omitted for patients achieving axillary pathologic complete response during NACT who are staged by TAD only. Nevertheless, we propose continuous follow-up evaluation for patients receiving TAD without completion ALND.

The strengths in our study design included a high degree of follow-up completeness and long follow-up times relating to the DBCG database as the data source. Furthermore, the TAD cohort in this study represented a consecutive cohort dating back to the start of TAD use in Denmark with prospectively collected follow-up data. By using left truncation, we accounted for the TAD cohort’s conditional survival until surgery, and with broad inclusion of competing events in the analysis, exhaustive outcome data are presented.

In addition, patients with cN2-3 disease were included in this study, although the possibilities for identifying this group for separate analysis are limited.

In conclusion, even with longer follow-up periods, RNR rates remain low for patients receiving TAD with ypN0 disease. Based on existing data, performing ALND for patients with ypN0 status found at TAD should be discouraged. The overall survival of these patients is excellent, providing necessary oncologic reassurance. Based on the available evidence, further studies may focus on additional patient groups that could be considered for further de-escalation of axillary surgery.

## Supplementary Information

Below is the link to the electronic supplementary material.

Supplementary file1 (DOCX 125 KB)

Supplementary file2 (DOCX 110 KB)

Supplementary file3 (DOCX 135 KB)

Supplementary file4 (DOCX 135 KB)

Supplementary file5 (DOCX 132 KB)
