# Supplementary material for: Oncologic First Events in Breast Cancer Patients After Targeted Axillary Dissection
Source: Ann Surg Oncol. 2025 Aug 20;32(13):9817–24. doi: 10.1245/s10434-025-18068-0 (PMC12589213; doi:10.1245/s10434-025-18068-0)
Supplement: Supplementary file 1 — Supplementary file1 (DOCX 125 KB) [file 10434_2025_18068_MOESM1_ESM.docx]

Supplemental Digital Content 1: Exclusion and truncation in a cohort of 17 534 node-negative patients used for analysis of oncological outcomes

15 784 patients for analysis

Abbreviations: SLNB: sentinel lymph node biopsy

596 patients with event or censoring before 201 days (312) or missing follow-up (284)

16 380 patients

1154 patients with previous malignancy

17 534 node-negative patients receiving primary SLNB
