# Supplementary material for: Oncologic First Events in Breast Cancer Patients After Targeted Axillary Dissection
Source: Ann Surg Oncol. 2025 Aug 20;32(13):9817–24. doi: 10.1245/s10434-025-18068-0 (PMC12589213; doi:10.1245/s10434-025-18068-0)
Supplement: Supplementary file 2 — Supplementary file2 (DOCX 110 KB) [file 10434_2025_18068_MOESM2_ESM.docx]

| Supplemental Digital Content 2: Characteristics of 15 784 cN0 pN0 patients staged with SLNB with no metastases in SNs | | |
| --- | --- | --- |
|  | **n (%)** | ***P^a^*** |
| **Age** |  | <.0001 |
| <30 years | 36 (0.2) |  |
| 30-39 years | 383 (2.4) |  |
| 40-49 years | 1624 (10.3) |  |
| 50-59 years | 4050 (25.7) |  |
| 60-69 years | 5737 (36.3) |  |
| 70-79 years | 2940 (18.6) |  |
| ≥80 years | 1014 (6.4) |  |
| **Tumor size at diagnosis (US)** |  | <.0001 |
| <20 mm | 12095 (76.6) |  |
| ≥20, <50 mm | 3494 (22.1) |  |
| ≥50 mm | 194 (1.2) |  |
| Missing | 1 (<0.1) |  |
| **Estrogen receptor status** |  | <.0001 |
| Positive | 14093 (89.3) |  |
| Negative | 1678 (10.6) |  |
| Missing | 13 (<0.1) |  |
| **HER2 receptor status** |  | <.0001 |
| Positive | 1529 (9.7) |  |
| Negative | 14215 (90.1) |  |
| Missing | 40 (0.3) |  |
| **Breast surgery** |  | <.0001 |
| Mastectomy | 2852 (18.1) |  |
| Breast conserving surgery | 12932 (81.9) |  |
| **Year of diagnoses** |  | NR^b^ |
| 2012-2013 | 3267 (20.7) |  |
| 2014-2015 | 3406 (21.6) |  |
| 2016-2017 | 3173 (20.1) |  |
| 2018-2019 | 3141 (19.9) |  |
| 2020-2021 | 2797 (17.7) |  |

^a^Compared to the demographic characteristics from the TAD group shown in table 1

^b^Not reported due to differences in group selection criteria
