# Supplementary material for: Oncologic First Events in Breast Cancer Patients After Targeted Axillary Dissection
Source: Ann Surg Oncol. 2025 Aug 20;32(13):9817–24. doi: 10.1245/s10434-025-18068-0 (PMC12589213; doi:10.1245/s10434-025-18068-0)
Supplement: Supplementary file 3 — Supplementary file3 (DOCX 135 KB) [file 10434_2025_18068_MOESM3_ESM.docx]

Supplemental Digital Content 3

Cumulative incidence of regional nodal recurrence and competing events in a cohort of 15 784 cN0 patients staged with SLNB with no SN metastases


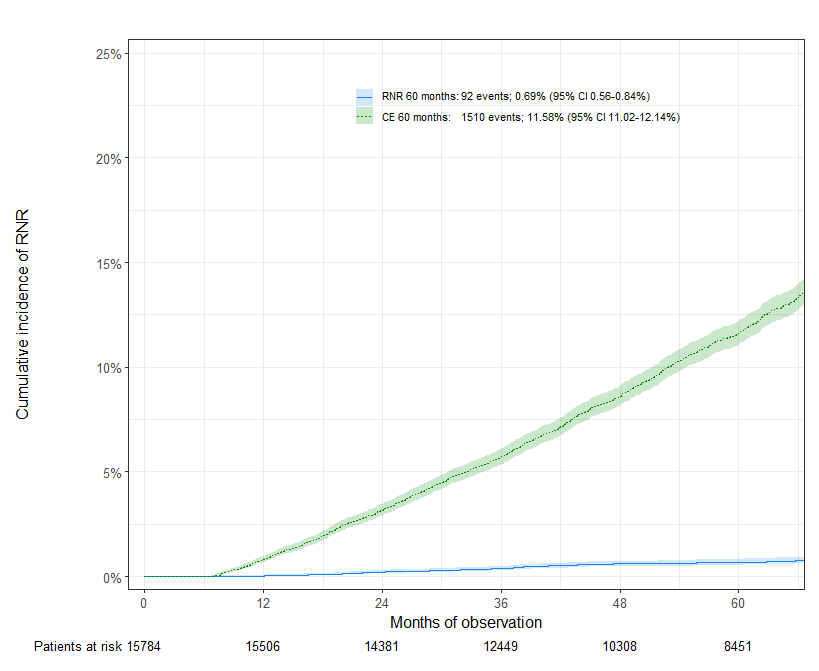


Abbreviations: SLNB: sentinel lymph node biopsy, RNR: regional nodal relapse, CE: competing event, CI: confidence interval
