# Supplementary material for: Oncologic First Events in Breast Cancer Patients After Targeted Axillary Dissection
Source: Ann Surg Oncol. 2025 Aug 20;32(13):9817–24. doi: 10.1245/s10434-025-18068-0 (PMC12589213; doi:10.1245/s10434-025-18068-0)
Supplement: Supplementary file 4 — Supplementary file4 (DOCX 135 KB) [file 10434_2025_18068_MOESM4_ESM.docx]

Supplemental Digital Content 4

Cumulated incidence of 1602 first events^*^ occurring in cN0 ypN0 patients within 5 years of follow-up


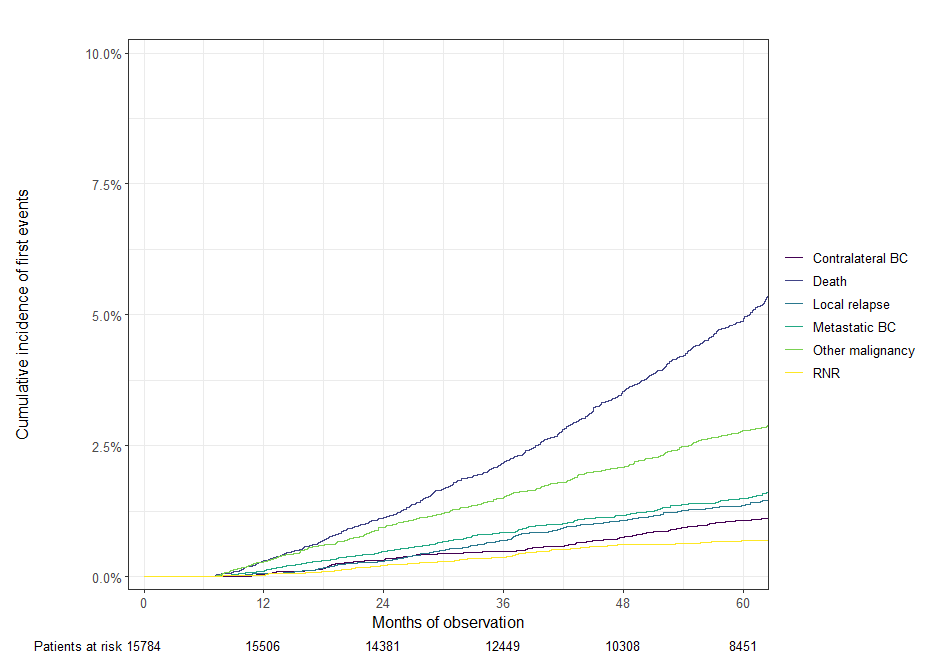


^*^Among patients with RNR (n = 92), isolated RNR occurred in 48 patients, RNR with local relapse in 15 patients and RNR with synchronous metastatic breast cancer in 29 patients.
