# Supplementary material for: Oncologic First Events in Breast Cancer Patients After Targeted Axillary Dissection
Source: Ann Surg Oncol. 2025 Aug 20;32(13):9817–24. doi: 10.1245/s10434-025-18068-0 (PMC12589213; doi:10.1245/s10434-025-18068-0)

Supplemental Digital Content 5:

Overall survival (OS) in a cohort of 15 784 cN0 pN0 patients staged with SLNB


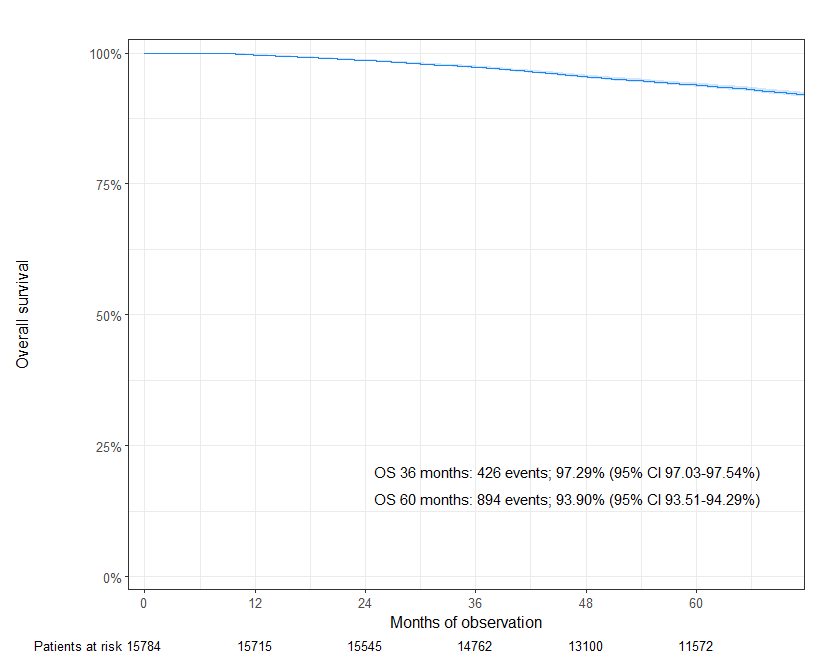

Supplement: Supplementary file 5 — Supplementary file5 (DOCX 132 KB) [file 10434_2025_18068_MOESM5_ESM.docx]
